# Complete and Assembled Genome Sequence of Bifidobacterium kashiwanohense PV20-2, Isolated from the Feces of an Anemic Kenyan Infant

**DOI:** 10.1128/genomeA.01467-14

**Published:** 2015-01-22

**Authors:** Pamela Vazquez-Gutierrez, Christophe Lacroix, Christophe Chassard, Jochen Klumpp, Christoph Jans, Marc J. A. Stevens

**Affiliations:** a Laboratory of Food Biotechnology, Institute of Food, Nutrition and Health, ETH Zürich, Zürich, Switzerland; b Laboratory of Food Microbiology, Institute of Food, Nutrition and Health, ETH Zürich, Zürich, Switzerland

## Abstract

The complete genome sequence of Bifidobacterium kashiwanohense strain PV20-2, an infant feces isolate, was determined using single-molecule real-time sequencing (SMRT). Hierarchical genome assembly resulted in a completely assembled genome of 2,370,978 bp. The B. kashiwanohense PV20-2 genome is the first completely sequenced and assembled genome of the species.

## GENOME ANNOUNCEMENT

Bifidobacteria represent an important commensal group of bacteria, being among the first microbial colonizers of the infant gut (1). Bifidobacterium kashiwanohense was originally isolated from feces of a healthy Japanese infant and is closely related to Bifidobacterium pseudocatenulatum and Bifidobacterium catenulatum, species commonly encountered in the infantile gut (2). B. kashiwanohense strain PV20-2 was isolated from the feces of an anemic Kenyan infant obtained during an iron intervention study (3). B. kashiwanohense PV20-2 was functionally characterized and selected for its high siderophore activity and high iron internalization activity (4). Genomic DNA was prepared using a lysozyme/mutanolysine-based cell lysis and subsequent purification using the Wizard genomic DNA purification kit (Promega, Madison, WI, USA) (5). The genome was sequenced using 3 single-molecule real-time sequencing (SMRT) cells on a PacBio RS II (Pacific Biosciences, Menlo Park, CA, USA) at the Functional Genomics Center Zurich (Zurich, Switzerland). A total of 119,341 reads with a mean length of 4,390 bp were assembled into a single contig using the hierarchical genome-assembly process (6). The genome was automatically annotated using the NCBI Prokaryotic Genomes Automatic Annotation Pipeline and manually curated using annotations obtained through the Rapid Annotation using Subsystems Technology (RAST) platform (7). The genome of B. kashiwanohense PV20-2 consists of a single 2,370,978-bp circular molecule and comprises 58 tRNA genes and 5 rRNA operons. The G+C content of the genome is 56.1%, and a total of 1,875 protein coding sequences (CDS) were predicted.

B. kashiwanohense PV20-2 is the first completely sequenced and assembled genome of the species. The genome will contribute to the understanding of genus’ evolution and adaptation to infantile gut.

### Nucleotide sequence accession number.

The genome sequence of B. kashiwanohense PV20-2 was deposited at the GenBank under the accession no. CP007456.
